# Genomic Profiling of Driver Gene Mutations in Chinese Patients With Non-Small Cell Lung Cancer

**DOI:** 10.3389/fgene.2019.01008

**Published:** 2019-10-18

**Authors:** Hongxue Meng, Xuejie Guo, Dawei Sun, Yuebin Liang, Jidong Lang, Yingmin Han, Qingqing Lu, Yanxiang Zhang, Yanxin An, Geng Tian, Dawei Yuan, Shidong Xu, Jingshu Geng

**Affiliations:** ^1^Department of Pathology, Harbin Medical University Cancer Hospital, Harbin, China; ^2^Department of Medicine, Geneis (Beijing) Co., Ltd., Beijing, China; ^3^Department of Thoracic Surgery, Harbin Medical University Cancer Hospital, Harbin, China

**Keywords:** lung cancer, driver mutations, epidemiology, EGFR, personalized medicine

## Abstract

Worldwide, especially in China, lung cancer accounts to a major cause of mortality related to cancer. Treatment decisions mainly depend on oncogenic driver mutations, which offer novel therapeutic targets for anticancer therapy. However, studies of genomic profiling of driver gene mutations in mainland China are rare. Hence, this is an extensive study of these mutations in Non-small-cell lung cancer (NSCLC) Chinese patients. Comparison of driver gene mutations of lung adenocarcinoma with other races showed that the mutational frequencies were similar within the different East Asian populations, while there were differences between East Asian and non-Asian populations. Further, four promising candidates for druggable mutations of epidermal growth factor receptor (*EGFR*) were revealed that open up avenues to develop and design personal therapeutic approaches for patients harboring mutations. These results will help to develop personalized therapy targeting NSCLC.

## Introduction

Globally, lung cancer is the most frequent cause the mortality compared to other cancer types. Non-small-cell lung cancer (NSCLC) accounts for close to 85% to 90% of all lung cancer cases (Planchard et al., 2018). There are three types of NSCLC based on histopathology, including adenocarcinoma (ADC), squamous cell carcinoma (SCC) and large cell carcinoma (LCC) (Travis et al., 2015). Treatment strategies for NSCLC have been revolutionized since the identification of epidermal growth factor receptor (*EGFR*) activating mutations which predict response to EGFR tyrosine kinase inhibitors (TKIs) in 2004 (Lynch et al., 2004; Paez et al., 2004). Examples of such drugs are erlotinib and gefitinib that have been instrumental in patients in terms of the response and survival without a relapse (Mitsudomi et al., 2010; Rosell et al., 2012). Guidelines from clinical practice offer recommendations of an analysis of mutations in *EGFR* before the start of therapy of advanced NSCLC (D’Addario et al., 2009; Azzoli et al., 2010; Ettinger et al., 2018). To date, at least nine important driver mutations causing NSCLC have been described and several markers are already used for best treatment strategy selection. In this context, the pervasiveness and occurrence of these mutations are different across populations such as that of East Asians and the white with more mutations in *EGFR* and lesser mutations in Kirsten Rat Sarcoma Viral Proto-Oncogene (*KRAS*) (Kohno et al., 2015). With very little data in this regard from mainland China, a study that describes the pattern of driver mutations will facilitate personal medicine for NSCLC and on the design of clinical trials.

The identification of these mutations has been facilitated by the use of three-dimensional (3D) protein structures to analyze interactions between proteins found more in mutations associated with cancer as used by (Porta-Pardo et al., 2015) and (Engin et al., 2016). Hotspot3D (Niu et al., 2016) is another tool that analyzed 3D structures for spatial clusters or hotspots to later study putative variants and their functions. Such studies have shown the potential function and relevance of driver mutations in a clinical scenario.

The current work reports an inclusive set of driver mutations in a large set of probable NSCLC patients of Chinese origin. Several rarely reported mutations, including *EGFR* mutations (V742I, I789M, N842H) related with erlotinib, gefitinib, lapatinib, and *EGFR* mutation (S811C) related with afatinib were discovered.

## Materials and Methods

### Patient and Sample Collection

From July 2016 to October 2018, 5,003 patients with lung adenocarcinoma (3,243 tumor tissues and 1,760 blood samples) and 230 patients with lung squamous cell carcinoma (134 tumor tissue samples and 96 blood samples) from Harbin Medical University Cancer Hospital were subjected to enrollment in this work. Specimens from surgery or biopsies were fixed in formalin and embedded in paraffin (FFPE) to generate samples, while blood samples were collected in 10 ml cell-free DNA BCT tubes (Streck, Inc). While an informed consent in a written format following the Declaration of Helsinki was collected from all patients, all protocols were within the recommendations and framework of the Ethics Committee of the aforementioned hospital.

### DNA Extraction From Tumor Tissue and Plasma

GeneRead DNA FFPE Kit (Qiagen) was used for DNA extraction from the FFPE samples. In parallel, plasma was extracted by centrifugation in accordance with previous work (Diehl et al., 2008; Madic et al., 2012). Briefly, Streck tubes were centrifuged at 1,600 g for 10 min at 4°C within 3 h of the blood draw. Supernatants were further centrifuged at 16,000 g for 10 min at 4°C to remove debris. Plasma was harvested and stored at -80°C until use. QIAamp Circulating Nucleic Acid kit (Qiagen) was used to isolate circulating DNA. Quantification of DNA from both sets of samples was done using Qubit (Life Technologies) in accordance with instructions from the manufacturer.

### Screening Mutations

Screening of mutations was performed by targeted sequencing using the Lung Cancer Ten Genes Panel (Geneis Co.Ltd) along with the Accel-NGS 2S Plus DNA Library Kit (Swift Biosciences) and NextSeq CN500 Personal Genome Machine (Illumina). Lung Cancer Ten Genes Panel (Geneis Co.Ltd) was used to test mutations in the *EGFR* kinase domain, *KRAS*, *NRAS*, *PIK3CA*, *HER2* kinase, *BRAF*, as well as fusions of *ALK*, *ROS1*, *RET* along with Mesenchymal Epithelial Transition Proto-Oncogene *(MET)* amplifications. The average sequencing depth of 500X for tissue and 1,000X for blood samples was considered reliable. DNA samples were normalized to yield 100–250 ng input. Accel-NGS 2S Plus DNA Library Kit (Swift Biosciences) was used to prepare whole genome libraries and through a series steps including covaris shearing (ctDNA can skip this step), end-repair, A-base addition, barcoded adapter ligation, and PCR amplification. Qubit dsDNA HS Kit (Invitrogen) was used to quantify the libraries while 2100 (Agilent) was used to assess quality in accordance with instructions from the manufacturer. Capture probes with 5’ biotin were used to cause a specific pull-down of library samples with target sequences to achieve enrichment. The kits previously mentioned above were used to quantify and check the quality of the captured library while sequencing of templates was done on NextSeq CN500 in accordance to instructions from the manufacturer.

### Mutation and Statistical Analysis

Variant calling was done on the Lung Cancer Ten Genes Panel (Geneis Co. Ltd) from NextSeq CN500 sequencing was the BWA and FreeBayes software. The common clinical databases were used in this study, including PharmGKB, the Human Gene Mutation Database (HGMD), Clinvar, Cosmic, SNPedia, 1000genome, and dbSNP. A blinded approach was followed using the frequency threshold of ≥0.4% and ≥1% to call a mutation for ctDNA samples and tumor tissues analyzed, respectively.

### Mutational Data Collection and Hotspot3D Processing

More than 800 promising candidates were predicted by mutation-drug cluster and network analysis for druggable mutations by Hotspot3D (Niu et al., 2016). Here, the 3,243 tissues data of lung adenocarcinoma patients in China were collected and several rare mutations were then found by filtered in 800 potential druggable mutations of Hotspot3D. Droplet digital PCR was used to validate these potential driver gene mutations in our clinical cases.

## Results

### Distribution of Oncogenic Driver Mutations in Lung Adenocarcinoma Tissue Samples

**Figure 1:**
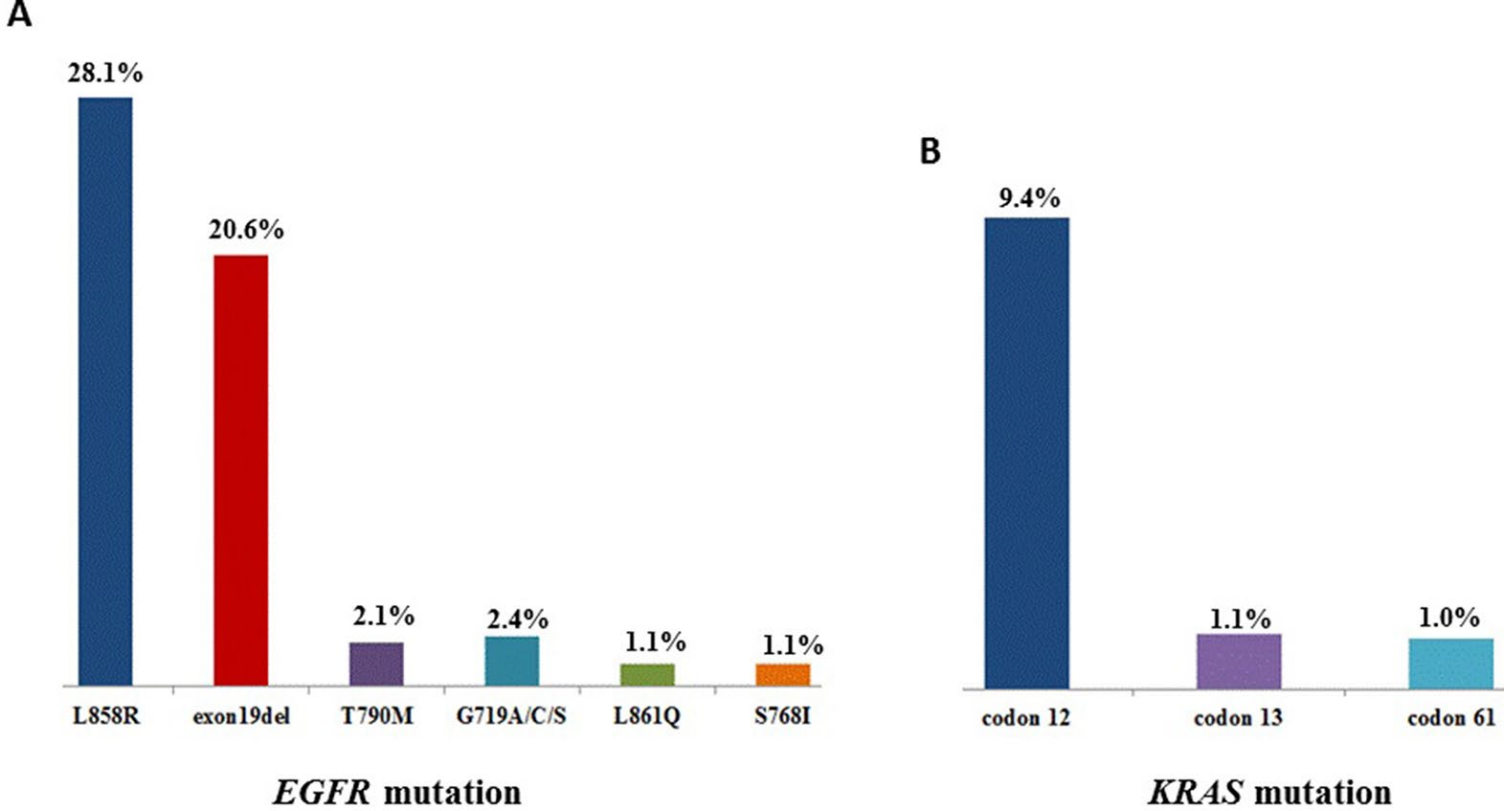
**(A)**, The mutation sites and frequency of *EGFR* and **(B)** *KRAS* in 3,423 patients with lung adenocarcinoma.

Sequencing of 3,243 tissues between July 2016 and October 2018 for oncogenic driver mutations was carried out. The distribution is as followed: mutations in *EGFR* kinase: 55.9%, *KRAS*: 11.7%, *NRAS*: 0.7%, *PIK3CA*: 2.9%, *HER2* (the analysis involved insertions in exon 20): 2.1%, *BRAF*: 1.6%. The next set are fusions of *ALK*: 2.8%, *ROS1*: 0.6%, *RET*: 0.6%, while that of *MET* amplifications was 1.3% (**Table 1**). As shown in **Figure 1**, 55.9% patients showed *EGFR* mutations, while the highest frequency of L858R was observed in 28.1% of the patients, followed by exon 19 deletion (20.6%). *KRAS* mutations were detected in 11.7% patients, with most of these were located in codon 12 (9.4%).

**Table 1:** Frequency of mutations in lung adenocarcinoma histologic subtypes.

Gene	Alteration	Frequency in NSCLC	Total frequency in NSCLC (n = 3243)
***ALK***	Rearrangement	2.8%	2.8%
***BRAF***	V600E	1.3%	1.6%
***EGFR***	Exon19del	20.6%	55.9%
	G719A/C/S	2.4%	
	L858R	28.1%	
	L861Q	1.1%	
	T790M	2.1%	
	S768I	1.1%	
***HER2***	Exon 20ins	2.1%	2.1%
***KRAS***	G12C/R/S/A/D/V	9.4%	11.7%
	G13C/R/S/A/D/V	1.1%	
	Q61K/L/R/H	1.0%	
***MET***	Amplification	1.1%	1.3%
***PIK3CA***	E542K	0.7%	2.9%
	E545K/Q	1.4%	
	H1047L/R	0.8%	
***NRAS***	G12C/R/S/A/D/V	0.3%	0.7%
	G13C/R/S/A/D/V	0.1%	
	Q61K/L/R/H	0.3%	
***RET***	Rearrangement	0.6%	0.6%
***ROS1***	Rearrangement	0.6%	0.6%

**Figure 2:**
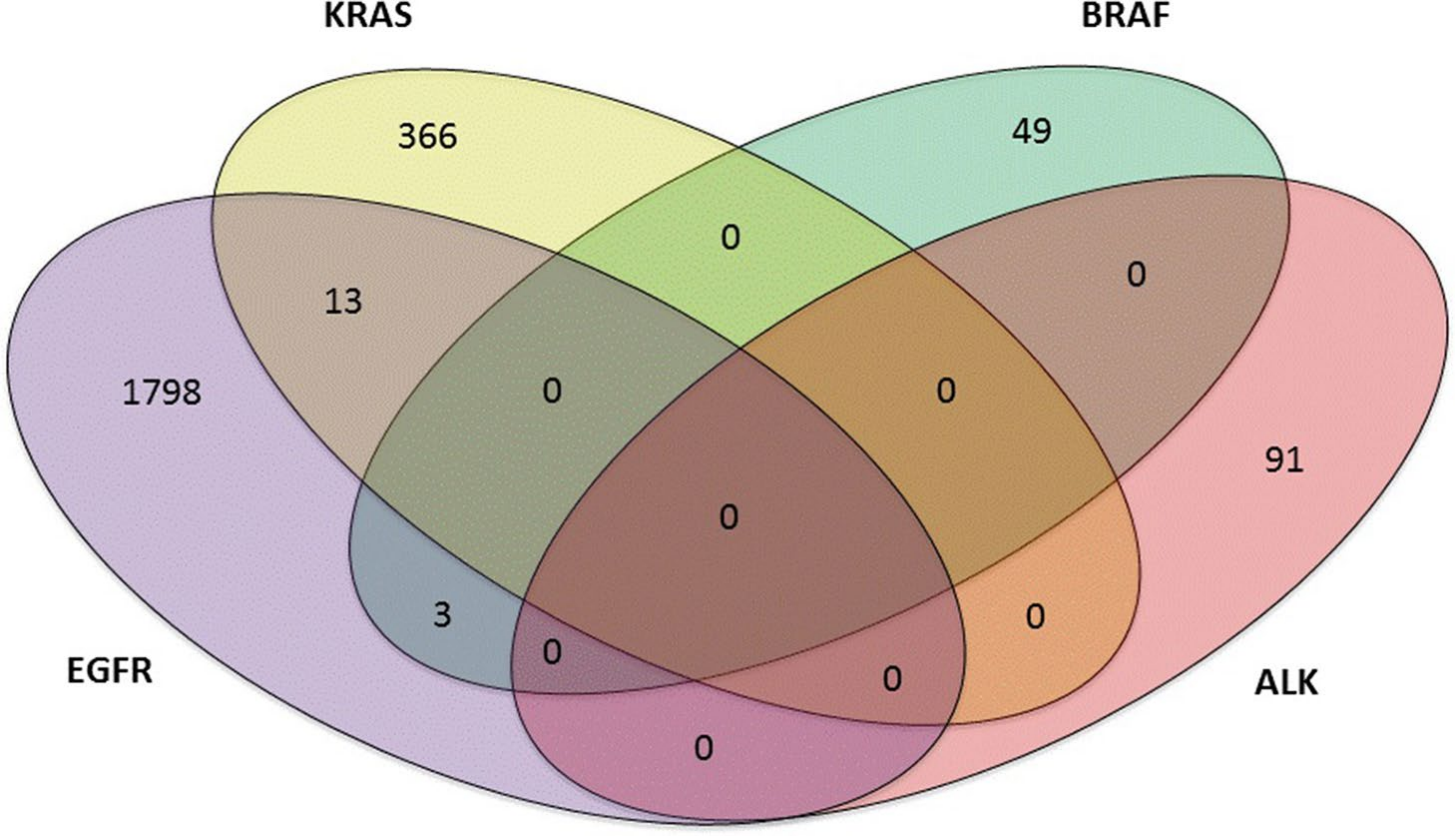
Four-set venn-diagram of single and multiple mutation panoramagram for lung adenocarcinoma tissue samples.

Of the 3,243 lung adenocarcinoma cases, 901 (901 out of 3,243, 27.8%) were negative, 2,185 (2,185 out of 3,243, 67.4%) harbored single mutations, and 157 (157 out of 3,243, 4.8%) were found to have multiple mutations. Mutations in *ALK*, *KRAS*, *BRAF*, and *EGFR* were studied. Thirteen patients coexisted *EGFR*+*KRAS* mutations; Three patients carried *EGFR*+*BRAF* mutations. However, *EGFR* and *ALK* mutations, *KRAS* and *BRAF* mutations, *KRAS* and *ALK* mutations, and *BRAF* and *ALK* mutations were mutually exclusive in our study (**Figure 2**). In addition, one patient carried a triple mutation: EGFR L858R + EGFR T790M + KRAS G12D, which was still rarely reported at present.

### Comparison of Driver Gene Mutations of Lung Adenocarcinoma With Other Races

Current research indicates that race plays a role in the genomics of NSCLC. To compare the frequency of driver mutations of lung adenocarcinoma with other races, we obtained the available data from several related studies (Serizawa et al., 2014; George et al., 2015; Yeung et al., 2015; Campbell et al., 2017). These results are summarized in **Table 2**. First, we found that the mutational frequencies were similar for black and white groups, but there were big differences between East Asian populations and non-Asian populations. Specifically, we found that *EGFR* was mutated at a much higher frequency in East Asian populations than in non-Asian populations (35.0-55.9% *vs* 11.6-14.4%). And it was the same for the most common mutations exon 19 deletions and exon 21 L858R. Another notable difference was that 33.5-34.2% of non-Asian patients had a *KRAS* mutation and this was significantly higher than the rate of 8.5-11.7% found for East Asians. In addition, *ALK* translocations are also important oncogenic drivers of NSCLC. It seems that *ALK* was mutated at a little higher frequency in East Asian populations than in non-Asian populations as presented in **Table 2**. But data from previous reports showed that *ALK* mutation frequencies were similar (3-5% *vs* 3-6%) between patients in East Asia (Japan, Korea, and China) and from those of European descent (Kohno et al., 2015). Further, overall mutational frequencies and copy number changes were not significantly different between mainland China (this study), Hong Kong, and Japan populations in lung adenocarcinoma. And no significant difference was observed in *BRAF*, *HER2*, *MET*, *PIK3CA*, *NRAS*, *RET*, and *ROS1* mutation status.

**Table 2:** Comparison of driver gene mutations of lung adenocarcinoma between mainland China (this study), Hong Kong (Diehl et al., 2008), Japan (Madic et al., 2012), Black, and White (George et al., 2015).

		Mainland China (3243)	Hong Kong (149)	Japan (411)	Black (146)	White (167)
		Mutant (%)	Mutant (%)	Mutant (%)	Mutant (%)	Mutant (%)
***ALK***	Rearrangement	2.8%	6.0%	5.0%	0.7%	0%
***BRAF***	V600E	1.3%	1.3%	0.7%	0.7%	1.2%
	Exon19del	20.6%	22.8%	–^a^	6.8%	6.0%
***EGFR***	L858R	28.1%	16.8%	–^a^	3.4%	4.2%
	Total	55.9%	43.0%	35.0%	11.6%	14.4%
***HER2***	Exon 20ins	2.1%	0.7%	1.7%	1.4%	0.6%
***KRAS***	G12/G13/Q61	11.7%	11.4%	8.5%	34.2%	33.5%
***MET***	Amplification	1.1%	1.3%	2.2%	2.1%	2.4%
***PIK3CA***	E542K/E545K/Q H1047L/R	2.9%	0.7%	2.7%	2%	2%
***NRAS***	G12/G13/Q61	0.7%	0.7%	0.5%	0%	1.2%
***RET***	Rearrangement	0.6%	–	1.1%	0%	1.2%
***ROS1***	Rearrangement	0.6%	2.0%	0.5%	0.7%	0%

a The mutation frequency was not mentioned in the related study.

### Distribution of Oncogenic Driver Mutations in Blood Samples of Patients

Sequencing of 1,760 blood samples (from July 2016 to October 2018) of patients revealed the following distribution: Mutations in *EGFR*: 32.6% patients, *KRAS*: 11.2% patients, *NRAS*: 1.0% patients, *PIK3CA* mutations: 2.9%, *HER2* kinase domain mutations: 0.9%, *BRAF*: 2.9% patients while *MET* amplifications were 0.7% (**Table 3**). It is noteworthy that the frequency of drug sensitive mutations, such as *EGFR* exon 19del and L858R, was reduced when compared with tissues (10.8% in blood and 20.6% in tissues for Exon 19del; 13.1% in blood and 28.1% in tissues for L858R). However, the frequency of drug resistant mutations, such as *EGFR* T790M, was increased when compared with tissues (5.1% in blood and 2.1% in tissues).

**Table 3:** Frequency of mutations in lung adenocarcinoma blood samples.

Gene	Alteration	Frequency in NSCLC	Total frequency in NSCLC (n = 1760)
***BRAF***	V600E	1.0%	1.4%
***EGFR***	Exon 19del	10.8%	32.6%
	G719A/C/S	1.4%	
	L858R	13.1%	
	L861Q	0.9%	
	T790M	5.1%	
	S768I	0.7%	
***HER2***	Exon 20ins	0.9%	0.9%
***KRAS***	G12C/R/S/A/D/V	5.9%	11.2%
	G13C/R/S/A/D/V	1.4%	
	Q61K/L/R/H	1.6%	
***MET***	Amplification	0.5%	0.7%
***PIK3CA***	E542K	0.8%	2.9%
	E545K/Q	1.2%	
	H1047L/R	0.9%	
***NRAS***	G12C/R/S/A/D/V	0.2%	1.0%
	G13C/R/S/A/D/V	0.3%	
	Q61K/L/R/H	0.5%	

### Frequency of Oncogenic Driver Mutations in Squamous Cell Carcinoma of Lung

Targeted DNA sequencing of 230 lung squamous cell carcinoma Chinese patient samples was done. Among those, there were 134 tissue samples and 96 blood samples, and 107(107 out of 134, 79.9%) and 74 (74 out of 96, 77.1%) were negative, respectively. In 134 lung squamous cell carcinoma tissue samples, there were 7 (5.2%) *EGFR* mutations, 6 (4.5%) *KRAS* mutations, 12 (9.0%) *PIK3CA* mutations, 1 (0.7%) *BRAF* mutations and 1 (0.7%) *MET* amplifications. In 96 lung squamous cell carcinoma blood samples, there were 8 (8.3%) *EGFR* mutations, 7 (7.3%) *KRAS* mutations, 6 (6.3%) *PIK3CA* mutations and 1 (1.0%) *BRAF* mutations. No *MET* amplifications were detected (**Table 4**). Our data adds confirmation with earlier work that lung squamous cell carcinoma shows a rare presence of two ubiquitous mutations seen in lung adenocarcinomas, *KRAS* and *EGFR*, are rare in lung squamous cell carcinoma (Ding et al., 2008). It is noteworthy that the rate of mutation of *PIK3CA* in these samples is relatively higher when compared with lung adenocarcinoma.

**Table 4:** Frequency of mutations in lung squamous cell carcinoma samples.

Gene	Alteration	Frequency in SCC tissues	Total frequency in SCC tissues (n = 134)	Frequency in SCC blood	Total frequency in SCC blood (n = 96)
***BRAF***	V600E	0.7% (1/134)	0.7%	1.0% (1/96)	1.0%
***EGFR***	Exon 19del	1.5% (2/134)	5.2%	2.1%(2/96)	8.3%
	L858R	3.7% (5/134)		6.3% (6/96)	
***KRAS***	K117N	0.7% (1/134)	4.5%	/	7.3%
	G12C/D/V	3.0% (4/134)		3.1% (3/96)	
	Q61H	/		1.0% (1/96)	
	A146T/P	0.7% (1/134)		3.1% (3/96)	
***MET***	Amplification	0.7% (1/134)	0.7%	/	/
***PIK3CA***	E542K	2.2% (3/134)	9.0%	1.0% (1/96)	6.3%
	E545Q/K	6.0% (8/134)		5.2% (5/96)	
	H1047R	0.7% (1/134)		/	

### *EGFR* Candidate Druggable Mutations Were Discovered by Filtered in Hotspot3D Results

**Figure 3:**
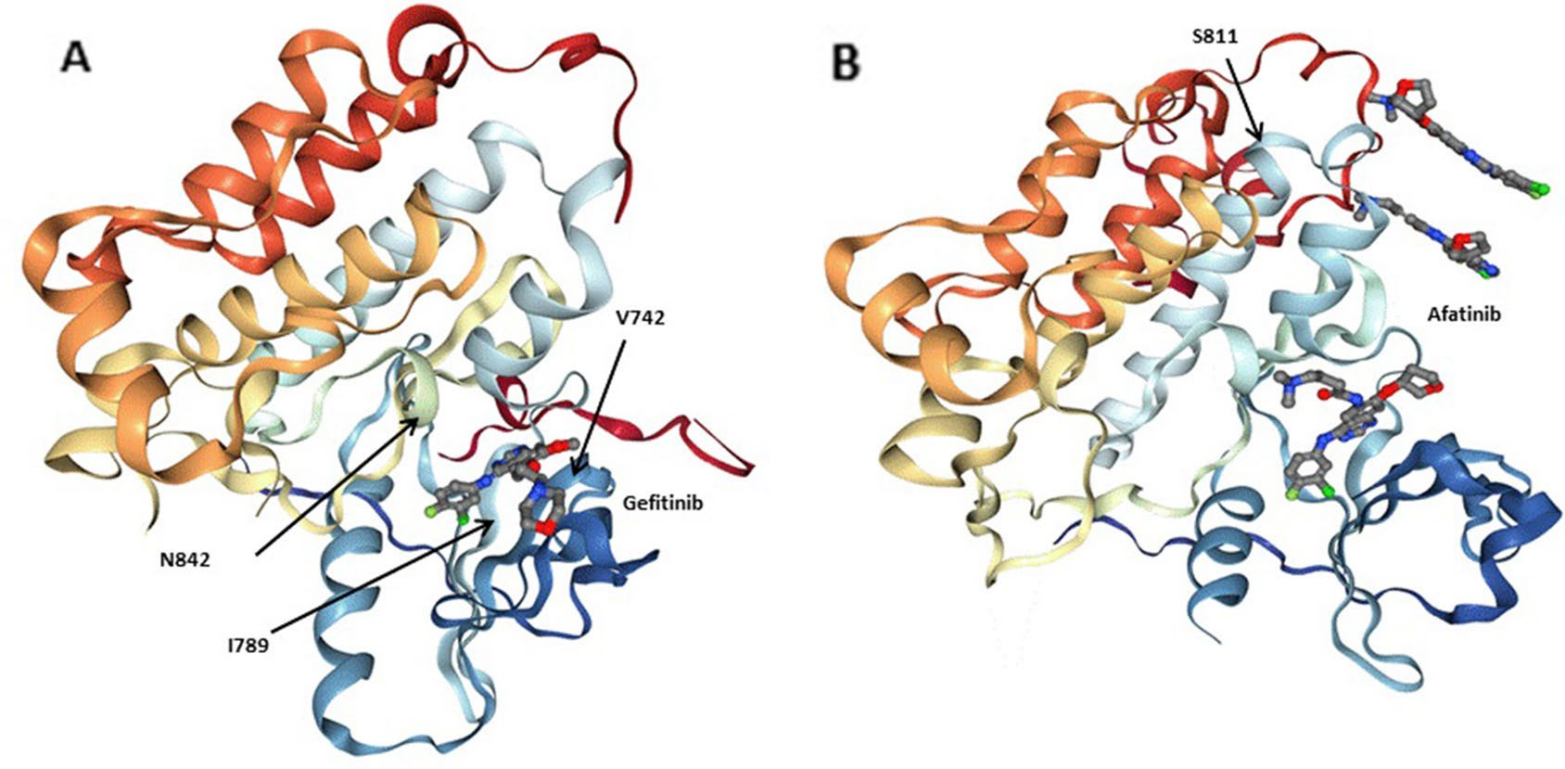
Three-dimensionional (3D) models of **(A)** the EGFRQ. kinase domain-Gefitinib complex structure (PDB: 2ITY), **(B)** the EGFR kinase domain-Afatinib complex structure (PDB: 4G5J). Gefitinib and Afatinib are shown as sticks. Residues at the mutation site of the EGFR kinase domains (V742, I789, N842 and S811) are shown with arrows.

We first collected the 3,243 tumors data of lung adenocarcinoma patients in China and several rarely reported mutations, including *EGFR* mutations (V742I, I789M, N842H) related with erlotinib, gefitinib, lapatinib, and *EGFR* mutations (S811C) related with afatinib were discovered (**Table 5**, **Figure 3**) by filtered in 800 potential druggable mutations of Hotspot3D. Droplet digital PCR was used to validate these *EGFR* variants in our clinical cases. We noticed that these *EGFR* rare variants always coexist with some common mutations, which showed poor prognosis in previous studies. The mechanism is still unclear. Functional verification of these *EGFR* druggable mutations will be performed in subsequent work.

**Table 5:** Several new *EGFR* druggable mutations were discovered by Hotspot3D in patients with lung adenocarcinoma.

Gene	Related drugs	Alterations	
***EGFR***	erlotinib; gefitinib; lapatinib	p.V742I	c.G2224A
	erlotinib; gefitinib; lapatinib	p.I789M	c.C2367G
	erlotinib; gefitinib; lapatinib	p.N842H	c.A2524C
	afatinib	p.S811C	c.C2432G

## Discussion

Identification of oncogenic driver mutations in NSCLC has greatly promoted clinical use and development of targeted drugs. Previous genomic studies of Chinese lung adenocarcinoma have not adequately represented patients. The current work involved a sizeable Chinese NSCLC patient sample set subjected to comprehensive investigation for driver mutations described as oncogenic. Our results were comparable with that detected in previous studies in Chinese lung adenocarcinoma (Gou and Wu, 2014), while the difference is mainly manifested in the different detection frequencies of several fusion genes. We suspected that was mainly due to the different platforms and detection methods. Comparison of driver gene mutations of lung adenocarcinoma with other races showed that the mutational frequencies were similar between mainland China (this study), Hong Kong, and Japan populations. But there were big differences between East Asian populations and non-Asian populations. Similar to Western population, the two most ubiquitous mutations were those in *EGFR* and *KRAS* in the case of lung adenocarcinoma samples. However, the *EGFR* mutation frequencies in East Asian lung adenocarcinoma were higher than previously reported in USA/Europe patients, whereas the overall frequency of *KRAS* mutations was much lower than in the West instead (D’Angelo et al., 2011; Smits et al., 2012; Kohno et al., 2015). A previous study found that Asians had the highest proportion of patients with mutations at 81% and the highest percentage of patients treated with targeted therapies (51%), while African Americans patients were the least likely to harbor mutations and to receive targeted therapy. However, there were no significant differences in overall survival between the four race groups (Steuer et al., 2016). A large dataset is still needed to verify this conclusion.

Although the *EGFR* S768I mutation is considered to be a very rare mutation, we detected a total of 1.1% patients with lung adenocarcinoma harboring this mutation. Due to its rarity and the variability of responses of treated cases, its exact function in TKI therapy is still not fully understood (Asahina et al., 2007; Masago et al., 2010). Subjects carried *BRAF* mutations with percent of 1.6%, and most of them were a V600E mutation. In addition, our data showed that *KRAS* and *BRAF* V600E mutations are mutually exclusive, which is in lieu of previous studies (Rajagopalan et al., 2002; De Roock et al., 2010). The stimulus to cancer development from both these genes is termed as, equivalent or at least redundant. In addition, *EGFR* and *ALK* mutations, *KRAS* and *ALK* mutations, and *BRAF* and *ALK* mutations were mutually exclusive in our study. Previous studies showed that *KRAS* mutations seem to be incompatible with *EGFR* mutations, but 13 cases of *KRAS* and *EGFR* coexisting mutations were found in our study, which means that the therapeutic effect of EGFR-TKIs in these samples would be ineffective. We also found three cases of simultaneous mutations of *EGFR* along with *BRAF*, which was first found in Li ‘study (Li et al., 2014). Interestingly, a triple mutation *EGFR* L858R+*EGFR* T790M+*KRAS* G12D was identified in our study, which was rarely reported at present. Clinical follow up was necessary for future researches.

1,760 lung adenocarcinoma patient blood samples were tested for analyzing mutations in *EGFR*, *KRAS*, *NRAS*, *PIK3CA*, *HER2*, *BRAF* and *MET* in cfDNA. The distribution of drug sensitive mutations, such as *EGFR* exon19del as well as, L858R, was decreased in comparison with these mutation in tissues, while the frequency of drug resistant mutations, such as *EGFR* T790M, was increased. It is speculated that may be related to the patient population. The majority of the patients analyzed with tumor tissues were to find targeted agents for the first time, while some of the patients analyzed with blood samples showed the presence of resistance developed towards EGFR-TKIs. It can be seen from the mutation frequency of *EGFR* T790M (2.1%), which was close to the *de novo* T790M frequency reported in the literature (Su et al., 2012). Almost all NSCLC patients administered therapy using EGFR-TKIs gradually manifest resistance. It is a recommendation nowadays to analyze such samples to check for the reason behind the resistance in these patients. Yet, a challenge here is mutations that underlie the disease in the case of advanced stages may not be entirely reflected in one sample biopsy particularly if the cancer is heterogeneous. Analysis of cfDNA or fragments of DNA minus cells can be an alternative to tissue samples as these fragments are released by apoptotic or necrotic cells with the level of these molecules correlated with the stage of the tumor and its prognosis (Diaz and Bardelli, 2014).

Studies have mainly involved adenocarcinoma in the case of NSCLC with molecular profiling of tumors capable of improving the outcome if therapies are targeted. However, such an approach fails in the case of SCC’s accounting for approximately 30% of all NSCLC. Here, we screened 230 Chinese patient samples of lung SCC and reported the rarity of two most ubiquitous mutations in *KRAS* and *EGFR* seen in lung adenocarcinoma, while *PIK3CA* mutations were relatively high when compared with lung adenocarcinoma. Most of the mutations are unknown in lung SCCs and it needs further research.

Besides this, we made a profound analysis of the 3,243 tumors data of lung adenocarcinoma patients in China, then three *EGFR* mutations (V742I, I789M, N842H) related with erlotinib, gefitinib, lapatinib, and one *EGFR* mutation (S811C) related with afatinib were discovered by filtered in Hotspot3D results. Next, we will continue to validate the function of the four *EGFR* rare druggable mutations by the following methods: (i) to predict of drug interaction based on protein structures; (ii) to perform biological validation in cultured cells; (iii) to establish the feasibility evaluation of clinical significance of these mutations by follow-up patients had these four *EGFR* rare mutations. Interestingly, we found that these *EGFR* rare variants always coexist with some common activating mutations in clinical samples. Whether this phenomenon has specific clinical significance needs to be further analyzed. Our analysis lends weight to novel approaches to address the use of personal medicine in patients with particular genetics.

In conclusion, we present a clear panoramagram of mutation frequencies of driver mutations in a sizeable population of NSCLC patients from China. There was an identification of four rare mutations in *EGFR* in these patients, such results can raise new possibilities for designing personalized treatments for patients carrying these mutations.

## Conclusion

Genomic profiling of driver gene mutations of a sizeable Chinese patient set with NSCLC was performed. Four promising candidates for druggable mutations of *EGFR* were revealed, which opens up new avenues in the development of therapies that target individual patients carrying such genetic alterations. These results will help to develop personalized therapy targeting NSCLC.

## Data Availability Statement

The data generated in this study can be found at http://www.ncbi.nlm.nih.gov/SNP/snp_viewTable.cgi?handle=DPSEQ_SNP

## Ethics Statement

This study was carried out in accordance with the recommendations of Ethics Committee of Affiliated Tumor Hospital of Harbin Medical University with written informed consent from all subjects. All subjects gave written informed consent in accordance with the Declaration of Helsinki. The protocol was approved by the Ethics Committee of Affiliated Tumor Hospital of Harbin Medical University.

## Author Contributions

HM and XG designed the study; HM, XG, DS, YH, and YA collected the data; YL and JL analysed the data; XG and DY interpreted the data; XG wrote the draft; YZ, QL, SX, and GT edited the manuscript; JG acquired the funding and supervised the whole study.

## Conflict of Interest

Authors XG, YL, JL, YH, QL, YZ, YA, GT, and DY were employed by company Geneis, China. The remaining authors declare that the research was conducted in the absence of any commercial or financial relationships that could be construed as a potential conflict of interest.
